# Using Smartphone Technology to Monitor Physical Activity in the 10,000 Steps Program: A Matched Case–Control Trial

**DOI:** 10.2196/jmir.1950

**Published:** 2012-04-20

**Authors:** Morwenna Kirwan, Mitch J Duncan, Corneel Vandelanotte, W Kerry Mummery

**Affiliations:** ^1^Centre for Physical Activity StudiesInstitute for Health and Social Sciences ResearchCQUniversityNorth RockhamptonAustralia; ^2^Faculty of Physical Education and RecreationUniversity of AlbertaEdmonton, ABCanada

**Keywords:** smartphone, health behavior, physical activity, matched case-control study, intervention

## Abstract

**Background:**

Website-delivered physical activity interventions are successful in producing short-term behavior change. However, problems with engagement and retention of participants in these programs prevent long-term behavior change. New ways of accessing online content (eg, via smartphones) may enhance engagement in these interventions, which in turn may improve the effectiveness of the programs.

**Objective:**

To measure the potential of a newly developed smartphone application to improve health behaviors in existing members of a website-delivered physical activity program (10,000 Steps, Australia). The aims of the study were to (1) examine the effect of the smartphone application on self-monitoring and self-reported physical activity levels, (2) measure the perceived usefulness and usability of the application, and (3) examine the relationship between the perceived usefulness and usability of the application and its actual use.

**Methods:**

All participants were existing members of the 10,000 Steps program. We recruited the intervention group (n = 50) via email and instructed them to install the application on their smartphone and use it for 3 months. Participants in this group were able to log their steps by using either the smartphone application or the 10,000 Steps website. Following the study, the intervention group completed an online questionnaire assessing perceived usability and usefulness of the smartphone application. We selected control group participants (n = 150), matched for age, gender, level of self-monitoring, preintervention physical activity level, and length of membership in the 10,000 Steps program, after the intervention was completed. We collected website and smartphone usage statistics during the entire intervention period.

**Results:**

Over the study period (90 days), the intervention group logged steps on an average of 62 days, compared with 41 days in the matched group. Intervention participants used the application 71.22% (2210/3103) of the time to log their steps. Logistic regression analyses revealed that use of the application was associated with an increased likelihood to log steps daily during the intervention period compared with those not using the application (odds ratio 3.56, 95% confidence interval 1.72–7.39). Additionally, use of the application was associated with an increased likelihood to log greater than 10,000 steps on each entry (odds ratio 20.64, 95% confidence interval 9.19–46.39). Linear regression analysis revealed a nonsignificant relationship between perceived usability (*r *= .216, *P *= .21) and usefulness (*r *= .229, *P *= .17) of the application and frequency of logging steps in the intervention group.

**Conclusion:**

Using a smartphone application as an additional delivery method to a website-delivered physical activity intervention may assist in maintaining participant engagement and behavior change. However, due to study design limitations, these outcomes should be interpreted with caution. More research, using larger samples and longer follow-up periods, is needed to replicate the findings of this study.

## Introduction

Physical inactivity has been identified as the fourth-leading risk factor for global mortality, causing an estimated 3.2 million deaths annually worldwide [1]. Regardless of the widespread understanding of the benefits of a physically active lifestyle, globally 60% of the population are considered insufficiently active to receive any health benefits [2]. In an attempt to reduce rates of physical inactivity, many behavioral modification programs have been developed [3-5]. Health promotion researchers have been quick to capitalize on the exponential growth of the Internet, and over the past decade an increasing number of interventions have been delivered online [6-11]. There is substantial evidence that online physical activity promotion programs are successful in producing short-term behavior change [12-14]. However, problems with engagement and retention of participants in online physical activity programs have been cited as an important issue preventing long-term behavior change [15]. In a review of website-delivered physical activity interventions, attrition ranged from 7% to 69%, with 9 of the 15 studies having an attrition of greater than 20% [15]. Several studies in this review also reported low exposure to intervention materials, due to a decline in website logins as the intervention progressed [15]. For example, in one study, website visits significantly declined over the intervention; 77% of all hits on the website were recorded in the first 2 weeks of the 8-week intervention [16]. It has been suggested that adjunctive delivery modes may enhance engagement in online physical activity interventions, which in turn may improve the effectiveness of the programs [17,18]. For example, the use of mobile phones in addition to an intervention website might be beneficial, as phones offer additional convenience and flexibility for the user, which may increase exposure to intervention materials [19].

Over the past two decades, mobile phones have evolved dramatically in both design and function, from simple call and text devices to the more sophisticated mini-personal computers known as smartphones. Mobile phones are more prevalent than computers or Internet access across the globe [20,21], with smartphones the fastest growing segment of the mobile handset market [22]. Unlike traditional mobile phones, smartphones allow individual users to install, configure, and run specialized applications of their choosing. At the end of 2010, over 17,000 smartphone health applications were available for consumers to download in major application stores [23]. It is estimated that 500 million people globally, out of a total of 1.4 billion smartphone users, will be using health-related smartphone applications by 2015 [23]. Despite the plethora of health- and fitness-related smartphone applications available, there is limited research into the effectiveness of these applications to promote health behavior change [24,25]. It should be noted, however, that technologies [26-28] relating to the self-monitoring of physical activity show promise, but these applications have yet to establish efficacy in terms of health behavior change. With consumers estimated to spend approximately a cumulative of 80 minutes per day using smartphone applications [29], there is a great potential for these devices to assist in health behavior change.

Some online physical activity programs have successfully included strategies to engage participants in self-monitoring behaviors as a means of increasing and maintaining activity levels [30-32]. One such program is 10,000 Steps (www.10000steps.org.au). 10,000 Steps is a freely accessible, ongoing, nonprofit, online physical activity health promotion program that encourages the use of step-counting pedometers to monitor daily physical activity levels [33,34]. With over 143,500 members [35] the 10,000 Steps program offers members multiple online interactive features to encourage participants to be active. One of the most prominent features of the website is the Step Log, where participants can record and monitor their daily physical activity levels [36]. The Step Log function on the 10,000 Steps website is the catalyst for members to record and monitor their activity levels. Evidence indicates that 10,000 Steps members engage with the website for approximately 44 days on average over their membership period [37]. Providing an alternative tool to log steps for the 10,000 Steps members, such as a smartphone application, may increase the duration of engagement in the intervention, due to the increased convenience such applications provide in eliminating the need to be at a computer to log steps. This may be important, as there is substantial evidence that the more frequently individuals engage with an online health intervention, the more likely they are to improve or maintain health-related behaviors [38]. To our knowledge this remains untested in regard to the self-monitoring of physical activity using both smartphone and website platforms.

Thus, the purpose of this study was to measure the effectiveness of a smartphone application, the iStepLog, to improve health behaviors in existing members of an online physical activity program (10,000 Steps, Australia). The aims of the study were to (1) examine the effect of the smartphone application on self-monitoring and self-reported physical activity levels, (2) measure the perceived usefulness and usability of the application, and (3) examine the relationship between the perceived usefulness and usability of the application and its actual use.

## Methods

### Participants

In a first step to test the potential of the iStepLog, we recruited intervention participants from the 10,000 Steps program, via an email (n = 6067) to members who had remained engaged with the intervention over a period of time (logged steps on at least one occasion in the 3 months preintervention). A total of 91 individuals responded, of whom 50 (24 women) met the inclusion criteria of having access to an iPhone or iPod touch for the duration of the study, as the iStepLog application was designed for the Apple platform. The study design was a 2-arm matched case–control trial. Intervention participants were matched to a control group (n = 150) of current 10,000 Steps members who were comparable in age, gender, length of membership, and average number and frequency of steps logged for the 3 months immediately preintervention. We specifically chose these matching characteristics, as they are potential confounding variables when comparing the two groups.

### Application

**Figure 1:**
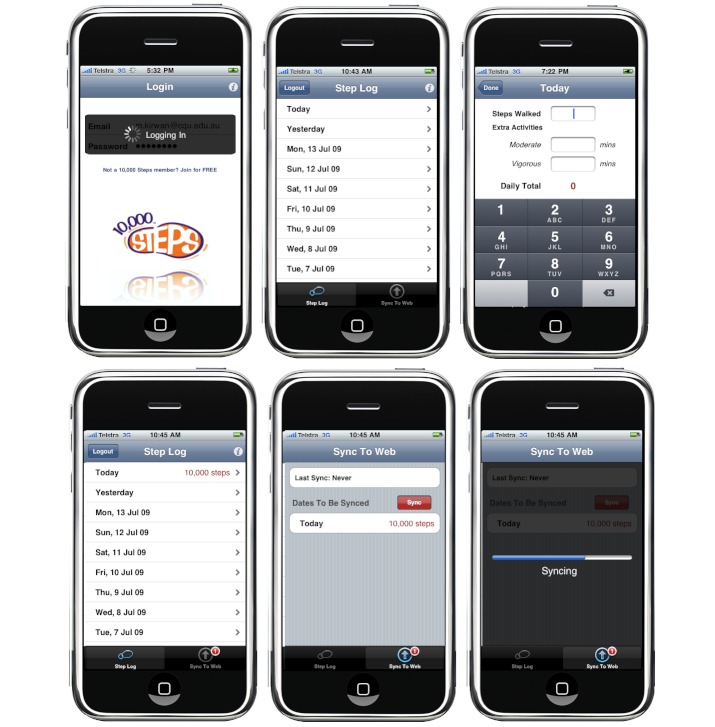
Design of iStepLog application. Top, left to right: login screen, step log screen, enter steps screen. Bottom, left to right: review steps screen, sync to Web screen, syncing screen.

The iStepLog application was designed to allow members of the 10,000 Steps program to record their daily physical activity levels on their mobile device and synchronize this information with their online Step Log (see Figure 1). Built-in tracking software was integrated into the application to allow researchers to monitor how much time participants spent using the application, how often they used the application to log steps, and which features of the application were most popular. Prior to this research, 10,000 Steps staff conducted a laboratory-based usability study using qualitative and quantitative measures to systematically improve the usability of the design of the iStepLog application. The outcomes of this research revealed that improvements to the iStepLog application in both aesthetic design and functionality resulted in increased performance of the application, in terms of both efficiency of use and a decreased number of problems experienced by users.

### Procedures

**Figure 2:**
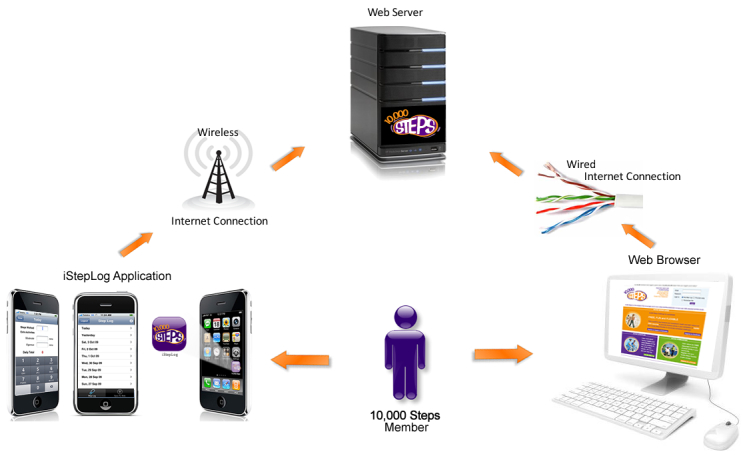
Procedure for intervention participants to upload their daily steps to the 10,000 Steps Web server.

After providing informed consent, the intervention group received the iStepLog application, to use on their own iPhone or iPod touch for 3 months. Participants in the intervention group were emailed an attachment with the iStepLog application, with instructions on how to install it on their smartphone. Over the course of the 3-month intervention (August to October 2009), these participants were able to log their steps either by using the iStepLog application on their device or by using the 10,000 Steps website (see Figure 2). Intervention participants were free to use either technology as they preferred; we did not require participants to continue to use the website during the intervention. Immediately following the study, we asked the intervention participants to complete a 10-item questionnaire concerning the usability and usefulness of the iStepLog application (see Table 1).

We selected matched group participants retrospectively, with the primary investigator blinded to the study period data and a strict protocol adopted. The matching procedure was performed for each intervention participant. The protocol followed was to first identify those individuals comparable in age (within 1 year either side) and of the same gender of the intervention participant. Of these individuals, we then isolated a cohort of possible matches. Possible matches had to be comparable for the number of steps logged each month (within 1000 steps either side) for the 3 months preintervention, as well as the frequency at which steps were logged each month (within one entry either side). From this selection, we chose the 3 individuals who most closely matched the length of time the intervention participant had been a member of 10,000 Steps. Matched group participants did not have access to the iSteplog application until after the completion of the study, when the application was officially launched in November 2009 and became available for dowload in Apple's online store (as of March 2012, more than 20,000 downloads of the application have been registered by Apple iTunes). On joining the 10,000 Steps program, the matched group participants had provided consent for their data to be used for research purposes. Ethics approval was gained from CQUniversity Human Research Ethics Committee prior to commencement.

### Measures

Data concerning participants’ usage of the iStepLog application and 10,000 Steps website were collected. This information included total number of steps logged over the period of the intervention; total number of days steps were logged; total number of days steps were logged via the application compared with a Web browser; and time spent using the iStepLog application on each occasion over the intervention period. As the iStepLog was not designed to automatically monitor steps, participants self-reported their step count and may have used a 10,000 Steps-supported pedometer (Yamax Digiwalker; Yamasa Tokei Keiki Co, Ltd, Tokyo, Japan), which is known to have high reliability [39] and validity [40], or they may have used a pedometer of another brand; we did not know the participants’ choice of pedometer.

When completing the usability and usefulness questionnaire, intervention participants rated their experience using a 5-point Likert scale (ranging from 1, strongly disagree to 5, strongly agree). The questions were based on a similar survey from a study evaluating the usability of the 10,000 Steps website [41].

### Statistical Analysis

We calculated descriptive statistics and means for all variables. The continuous variable of total number of days on which steps were logged was dichotomized to those participants who logged steps every day and those who did not log steps every day, regardless of the device used to log steps (computer or smartphone). We selected this dichotomization because it has been suggested that the daily monitoring of physical activity levels alone may be enough to facilitate a change in behavior [42]. Logistic regression analyses were conducted to calculate the odds ratios and 95% confidence intervals of the intervention group logging steps on a daily basis and recording over 10,000 steps on each entry, compared with the matched group. Measured by Likert scale data, subjective usability and usefulness survey items from the intervention group were reverse scored so that higher values represented more favorable responses to the questions. We performed factor analysis using principal components analysis and varimax rotation on the overall scale to identify factors within the overall scale. Factor analysis provided two definable factors with eigenvalues over 1.0, and all items had factor loadings of at least 0.5; both factors contained five questions each. One item (question 6) displayed significant cross-loading on both factors, and we determined that from a theoretical perspective this item aligns more closely with the second factor; we therefore included this item on the second factor for all subsequent analysis. Table 1 lists questions included in the first factor, defined as usability (questions 1 to 5). We defined the second factor (questions 6 to 10) as usefulness.

**Table 1:** Outcomes of the usability and usefulness questionnaire (n = 44), rated on a scale from 1 (strongly disagree) to 5 (strongly agree).

Questionnaire item			Rating, mean (SD)	Agree or strongly agree, n (%)
**Usability**				
	1	I think the iStepLog application is user-friendly	4.45 (0.66)	91% (40/44)
	2	I like the overall presentation and layout of the iStepLog application	4.24 (0.66)	89% (39/44)
	3	I was able to easily find my way around the iStepLog application	4.48 (0.59)	95% (42/44)
	4	I was able to easily enter and edit my steps in the application	4.39 (0.65)	91% (40/44)
	5	I was able to easily sync my steps to the 10,000 Steps website	4.21 (0.81)	80% (35/44)
Scale average of questions 1 to 5			4.35 (0.67)	89% (39/44)
**Usefulness**				
	6	It was convenient for me to use the iStepLog application	4.50 (0.70)	89% (39/44)
	7	I prefer to use the iStepLog application rather than go to the 10,000 Steps website to enter my steps	4.35 (0.75)	84% (37/44)
	8	The iStepLog encouraged me to log my steps more often than before it was available	4.00 (0.83)	80% (35/44)
	9	I didn’t visit the 10,000 Steps website as often because I used the iStepLog application	4.33 (0.76)	84% (37/44)
	10	I would like to continue using the iStepLog application	4.47 (0.70)	89% (39/44)
Scale average of questions 6 to 10			4.33 (0.75)	84% (36.8/44)

Usability and usefulness scores were calculated as the mean of scale items. We used separate linear regression models to examine the relationship between use of the application (dependent variable), in terms of total number of days participants logged steps, and the perceived usability and usefulness of the application (independent variables). We used PASW version 18.0 (IBM Corporation, Somers, NY, USA), with the significance level being set at alpha =.05.

## Results

Table 2 outlines the variables matched for both groups. The average age of intervention group participants was 39.3 (SD 12.8) years and covered a wide age range (17 to 64 years); and 48% (n = 24) were women. As the intervention sample was matched with 10,000 Steps participants with similar demographics, the average age in the matched group was 40.1 (SD 12.1) years and 48% (n = 72) were also women. Intervention and matched participants were matched on the amount of time they had been members of the 10,000 Steps program, ranging from 40 to 820 days. An independent *t *test revealed no significant difference in the duration of membership (*t*_198 _= 0.779, *P *= .44) between the intervention and matched participants.

Table 3 outlines the findings concerning use of the iStepLog application of the 10,000 Steps program. Over the study period, the frequency with which participants logged steps declined significantly in the matched group (61 days preintervention, 41 days intervention), compared with the intervention group, which maintained their frequency of logging (61 days preintervention, 62 days intervention). In the 3 months preintervention, both groups were logging on average 10,000 steps on each occasion. During the study period the intervention group maintained their step count, but the matched group logged a significantly lower number (mean 6274.73, SD 2106.11 steps, *P *< .001). Logistic regression analyses revealed that use of the application was associated with an increased likelihood to log steps on a daily basis during the intervention period compared with those not using the application (odds ratio 3.56, 95% confidence interval 1.72–7.39). Additionally, we found that the use of the application was associated with an increased likelihood to log greater than 10,000 steps on each entry (odds ratio 20.64, 95% confidence interval 9.19–46.39). Further analysis revealed that that the intervention group used the iStepLog application 71.22% (2210/3103) of the time to log steps when compared with a traditional Web browser (893/3103, 28.8%). Participants spent approximately 9 seconds using the application on each occasion.

**Table 2:** Matched variables for the intervention and matched groups.

	Intervention (n =50)	Matched (n = 150)	*P *value
Age (years), mean (SD)	39.3 (12.8)	40.1 (12.1)	.97
Gender (female), n (%)	24 (48%)	72 (48%)	
Length of membership (days), mean (SD)	426.79 (373.18)	430.31 (389.24)	.44
Steps logged per day in the 3 months preintervention, mean (SD)	10980.33 (3308.36)	10635.43 (3987.20)	.67
Total days steps logged 3 months preintervention, mean (SD)	60.90 (11.02)	61.30 (10.06)	.94

**Table 3:** Self-monitoring results for the intervention and matched groups over the 3-month intervention period.

	Intervention (n = 50)	Matched (n = 150)
Total days steps logged, mean (SD)	62.06 (12.48)	41.36 (12.25)*
Daily steps logged across study period, mean (SD)	11140.22 (4121.33)	6274.73 (2106.11)*
Ratio of steps logged using iStepLog application, n (%)	2210/3103 (71.22%)	NA^a^
Total time iStepLog application used per participant (minutes over intervention period), mean (SD)	11.1 (3.74)	NA^a^
Time per usage (seconds), mean (SD)	9.33 (3.21)	NA^a^

^a ^Not applicable.

Table 1 shows the mean scores for the 10 usefulness and usability questionnaire items. In the intervention group, 6 participants did not complete the final questionnaire. Over 80% of respondents reported either agreeing or strongly agreeing with each item, which resulted in a high mean overall score for usefulness (mean 4.33, SD 0.75) and usability (mean 4.35, SD 0.67). Internal consistency (Cronbach alpha) for the usability (alpha = .88) and usefulness questionnaire items (alpha = .88) was high, indicating acceptable reliability of the measure. Linear regression analysis on both factors revealed a nonsignificant relationship between perceived usability (*r *= .216, *P *= .21) and usefulness (*r *= .229, *P *= .17) of the application and frequency of logging steps within the intervention group.

## Discussion

This study examined the effectiveness of a smartphone application in increasing the frequency of self-monitoring of physical activity in active members of the online-delivered 10,000 Steps program. There is limited research measuring the usage of a health-related smartphone application and its effect on the behavior of users. The majority of health applications available for consumers are funded by commercial organizations, which are disinclined to distribute their usage information in a competitive market.

The iStepLog application was developed as an additional mode of delivery, to supplement the existing online method of interaction with the 10,000 Steps program. This research revealed that the iStepLog application assisted participants in maintaining engagement with the program. This is in contrast to the matched group, which had a significant decline in the frequency and number of steps logged over the intervention period. The difference in average daily steps between the groups is important from a health perspective because, according to established guidelines [43], the intervention group was considered to be active before the intervention and maintained this level of physical activity during the intervention period. Similarly, the matched group was considered to be active prior to the study period; however, this group declined to a level considered as somewhat active. The finding that the intervention group maintained their frequency of logging steps and the amount of activity over the study period is encouraging, as there is substantial evidence that the more frequently individuals engage with an online health intervention, the more likely they are to improve or maintain health-related behaviors [38]. Glasgow and colleagues [44] measured usage patterns of their diabetes self-management website and found that greater use of the website, and especially engagement in self-monitoring, was related to greater improvement in physical activity. Over the 4-month intervention period (112 days), diabetes patients logged their activity levels on average 53% of the time (59 days). In comparison, our study, conducted over 3 months (90 days), found that intervention participants logged their activity levels 69% of the time (62 days). The frequency with which participants logged steps was much higher in our study, and this may be attributed to the addition of the iStepLog application. Further research needs to be conducted to evaluate the long-term impact on health outcomes, incorporating objective measures of physical activity.

The high usage frequency (average of 40 times during the study period) and proportion of time (71% smartphone vs 29% website) the iStepLog application was used, and the high usability and usefulness scores suggest that intervention participants not only liked the design of the application, but also found it convenient and used it frequently. In our research, usability and usefulness of the iStepLog application were not significantly correlated with usage. This is likely due to the high usability and usefulness scores recorded across all intervention participants; this ceiling effect limits the variability in the scores to enable such a correlation.

Usage of the iStepLog application was high, when compared with a recent survey conducted by the Consumer Health Information Corporation, which found that smartphone applications have a high rate of dropouts, with 26% being used only once and 74% being discontinued by the 10th use [45]. This is encouraging, as it is established that for a user to adopt and frequently use a smartphone application they must consider it both usable and useful [46]. Considering that lack of usability and usefulness are top reasons for users to discontinue smartphone application usage [45], our findings illustrate how important it is not only to measure uptake and usage of smartphone applications, but also to consider measuring usability and usefulness, as this influences long-term adoption [47]. The average time spent using the application on each occasion in this study (9.3 seconds) is at the lower end of the range reported by others, ranging from 10 to 250 seconds [48]. In light of the intent of the iStepLog application to log steps and of the high usability scores in this study, we do not view this as a negative; rather it is consistent with the intent and design of the application to provide participants with a time-efficient mode of logging steps.

The effectiveness of smartphone applications to improve health behaviors is an emerging field of research. Abroms and colleagues [49] have examined the quality of the content provided in smoking cessation smartphone applications. They found that the 47 applications had low levels of adherence to established guidelines for smoking cessation [49]. Abroms and colleagues recommended that current applications be revised and all future applications be developed around evidence-based practices. With no current regulation of the health advice provided in smartphone applications, their recommendations are pertinent. Quality control is definitely a concern and very relevant considering the plethora of health applications (17,000) available for smartphone users [23]. Companies such as Apple have established guidelines for smartphone applications development, but these guiding principles relate solely to aesthetic design and functionality, and not to content. As Abroms and colleagues’ [49] research highlighted, many health-related smartphone applications are providing misinformation. Further research needs to be conducted to establish both the quality of information provided and the efficacy of these applications in improving consumer health.

Several limitations of this study should be noted. This study design was a matched case–control trial of short duration with a small sample size, and this limits the generalizability of the findings. Due to the low response rate and eligibility criteria (being a 10,000 Steps member and owning an iPhone), intervention group participants were not representative of the wider population. The intervention group was also self-selected and may have been more motivated than the matched group; however, we attempted to limit this by matching this group on key demographic variables. A further limitation is that intervention participants knew they were part of a research study and were given an innovative self-monitoring tool, and this may have influenced their behavior, whereas this was not the case in the control group. Concomitantly the matched group did not receive a stimulus, and this may explain their lack of engagement and interaction with the 10,000 Steps program over the study period. Despite these limitations, this research contributes to a paucity of work concerning smartphone applications and their use as a health promotion tool. The results of this study highlight the importance of continuing to evaluate the effectiveness of smartphone applications to influence health behaviors.

The outcomes of this study suggest that the use of a smartphone application to self-monitor physical activity may assist in maintaining an active lifestyle. Further, providing a smartphone application as an adjunct tool for the delivery of an online physical activity promotion program may assist with maintaining participant engagement. From this study we have gained insight into the potential of smartphone applications to improve health behaviors. However, due to study design limitations, the outcomes should be interpreted with caution. More research is needed to determine the long-term outcomes of adopting this third generation of wireless technology as a tool in health promotion. In particular, examining the effectiveness of the iStepLog application among new users not previously exposed to a physical activity intervention could provide important insight into the effectiveness of this technology in supporting behavior change.
